# Sevoflurane Induces Ferroptosis of Glioma Cells Through Activating the ATF4-CHAC1 Pathway

**DOI:** 10.3389/fonc.2022.859621

**Published:** 2022-03-17

**Authors:** Yingyi Xu, Na Zhang, Cheng Chen, Xinke Xu, Ailing Luo, Yaping Yan, Yanhua Lu, Jianhua Liu, Xinxu Ou, Yonghong Tan, Yufeng Liang, Lihe Chen, Xingrong Song, Xiaoping Liu

**Affiliations:** ^1^ Department of Anaesthesiology, Guangzhou Women and Children’s Medical Center, Guangzhou Medical University, Guangzhou, China; ^2^ Department of Neurosurgery, Guangzhou Women and Children’s Medical Center, Guangzhou Medical University, Guangzhou, China; ^3^ Department of Hematology, Guangzhou Women and Children’s Medical Center, Guangzhou Medical University, Guangzhou, China; ^4^ Operating Room, Guangzhou Women and Children’s Medical Center, Guangzhou Medical University, Guangzhou, China; ^5^ Pediatric Intensive Care Unit, Guangzhou Women and Children’s Medical Center, Guangzhou Medical University, Guangzhou, China; ^6^ Department of Pediatrics, Linzhi People’s Hospital, Linzhi, China; ^7^ Medical Library, Guangzhou Women and Children’s Medical Center, Guangzhou Medical University, Guangzhou, China

**Keywords:** glioma, sevoflurane, ferroptosis, ATF4, CHAC1

## Abstract

**Objective:**

To clarify the function and mechanisms of sevoflurane (Sev) on ferroptosis in glioma cells.

**Methods:**

Different concentrations of Sev were used to treat glioma cells U87 and U251. Ferroptosis inducer Erastin was used to incubate glioma cells combined with Sev and ATF4 siRNA transfection treatment. CCK-8 assay and colorimetric assay were performed to analyze cell viability and Fe^+^ concentration, respectively. The releases of reactive oxygen species (ROS) were determined by flow cytometry analysis. Transcriptional sequencing was used to screen the differential genes affected by Sev in U251 cells. The mRNA and protein expression of ferroptosis-associated genes was detected by qRT-PCR and Western blotting.

**Results:**

Sev could suppress cell viability, increase ROS levels and Fe^+^ concentration, downregulate the protein expression levels of GPX4, and upregulate transferrin, ferritin, and Beclin-1 in a dose-dependent manner in U87 and U251 cells. The expression of ferroptosis and mitophagy-related gene activating transcription factor 4 (ATF4) was identified to be enhanced by Sev analyzed by transcriptional sequencing. ChaC glutathione-specific gamma-glutamylcyclotransferase 1 (CHAC1), which is involved in ferroptosis, is a downstream gene of ATF4. Inhibition of ATF4 could interrupt the expression of CHAC1 induced by Sev in U87 and U251 cells. Ferroptosis inducer Erastin treatment obviously inhibited the cell viability, elevated the Fe^2+^ concentration, and promoted ROS generation in U87 and U251 cells. The protein level of ATF4 and CHAC1 was increased in Erastin-treated U87 and U251 cells. Moreover, the interruption of Sev-induced ferroptosis and CHAC1 activating induced by ATF4 suppression could be reversed by Erastin.

**Conclusions:**

In summary, this study suggested that Sev exposure-induced ferroptosis by the ATF4-CHAC1 pathway in glioma cells.

## Introduction

Glioma is the most frequently occurring primary malignant tumor in both children and adults with a median survival time of approximately 14 months (1, 2). The main characteristics of glioma including unlimited cellular proliferation, resistance to apoptosis, diffuse infiltration, and angiogenesis make it difficult for it to be completely removed by surgical resection as a first-line therapy (3, 4). Instead, temozolomide chemotherapy and radiotherapy are considered important treatment options for glioma. Unfortunately, a lower than 10% 5-year survival rate usually occurred, accompanied by instinct adverse effects in patients (5–7). Several factors such as the blood–brain barrier, tumor location in the brain, and gene variation influence the treatment effect of glioma (8). In addition, cellular metabolic disorders are signatures of gliomas and play important roles in the progression of gliomas (9). Thus, it is urgently needed to elucidate the mechanism underlying the initiation and development of glioma for developing novel targets for glioma therapy.

Ferroptosis has been recently recognized as an iron-dependent and atypical cell death form with the main features, including excessive lipid peroxidation products and lethal reactive oxygen species (ROS) (10, 11). It has been reported that ferroptosis is genetically and morphologically distinct from caspase-dependent apoptosis (12). Related studies have indicated that cellular ferroptosis could be induced after downregulation of glutathione peroxidase 4 (GPX4) (13) as well as upregulation of iron storage protein ferritin and iron-carrier protein transferrin (14). The majority of ferroptosis-related genes were differentially expressed among glioblastoma, low-grade glioma, and non-tumor brain tissue. Moreover, ferroptosis-related gene-related risk scores could predict glioma prognosis (15). Cheng et al. revealed that activating ferroptosis could suppress proliferation of glioma cells (16). Amentoflavone was reported to inhibit cell proliferation and promote cell death through inducing autophagy-dependent ferroptosis in glioma (17). Multiple anesthetics are used for neurosurgical operation. However, the effects of these anesthetics on glioma are still unclear.

Sevoflurane (Sev) as a volatile anesthetic which is commonly used in clinical operations has been recently reported to exert antitumor physiologic effects in several tumors, including breast cancer (18), lung cancer (19), and colon cancer (20). Accumulating evidence has indicated that Sev exerts suppressive effects on the proliferation and metastasis of glioma cells in different molecular mechanisms, including suppressing Rac1/paxillin/FAK and Ras/Akt/mTOR in several tumor cells (21), regulating the ANRIL/let-7b-5p axis (22), and depleting macrophages from the melanoma microenvironment (23). Sev could also inhibit glioma cell proliferation and metastasis through the miRNA-124-3p/ROCK1 axis (24), KCNQ1OT1/miR-146b-5p/STC1 axis (25), miR-34a-5p/MMP-2 axis (26), and circ_0002755/miR-628-5p/MAGT1 axis (27). Wu et al. identified that Sev disturbed iron homeostasis and caused iron overload in both *in vitro* hippocampal neuron culture and *in vivo* hippocampus (28). Base on the above studies, we speculated that Sev might function on ferroptosis in glioma cells.

In order to confirm the hypothesis, we firstly analyzed the effects of different concentrations of Sev on ferroptosis-associated iron accumulation, ROS accumulation, and expression of ferroptosis-related genes in two glioma cell lines. Then, we performed transcriptional sequencing to screen the differentially expressed genes regulated by Sev. Moreover, we used rescue experiments to illustrate the possible mechanism of Sev on glioma cells.

## Materials and Methods

### Chemicals and Reagents

DCFDA/H2DCFDA-Cellular ROS Assay Kit was provided by Abcam (ab113851, Cambridge, MA, USA). Erastin (HY-15763, ferroptosis inducer) was purchased from MedChemExpress (Shanghai, China). The following primary antibodies were used in this study: anti-ATF-4 antibody (ab184909, Abcam, USA), anti-CHAC1 antibody (MA5-26311, Invitrogen, Carlsbad, CA, USA), anti-GPX4 antibody (ab125066, Abcam, USA), anti-transferrin receptor antibody (ab277635, Abcam, USA), anti-ferritin antibody (ab75973, Abcam, USA), anti-Hsp70 antibody (ab2787), and anti-GAPDH antibody (ab8245, Abcam, USA). Goat anti-mouse IgG (HRP) (ab6789, Abcam, USA) and goat anti-rat IgG (HRP) (ab97057, Abcam, USA) were used as secondary antibody for Western blotting.

### Cell Lines and Treatment

Human glioma cell lines (U87 and U251) were provided by the Shanghai Institutes for Biological Sciences Cell Resource Center and grown in DMEM containing 10% FBS (Gibco, Grand Island, NY, USA) at 37°C containing 5% CO_2_. Then, U87 and U251 cells were treated with 1.7%, 3.4%, and 5.1% Sev gas for 2 h. Activating transcription factor 4 (ATF4) siRNA (sequences: 5′-GAGCCAATAAGAGCTCGAGATATAT-3′) or control sequences (5′-GAGTAAGAACGAGCTAGAATCCTAT-3′) were transfected to cells *via* Lipofectamine 2000 (Invitrogen, Foster City, CA, USA), followed by 5.1% Sev treatment for 2 h. In addition, U87 and U251 cells in the Sev-treated plus ATF4 siRNA-transfected group were treated with 10 μM Erastin for 24, 48, and 72 h.

### Cell Viability Assay

The Cell Counting Kit-8 (CCK-8, Beyotime, Beijing, China) was utilized to examine the cell viability of glioma cells according to the manufactures’ instructions. In brief, cells at a density of 3,000 cells per well from different groups were seeded into 96-well plates and cultured overnight. Next, cells in each well were incubated with 10 μl CCK-8 reagent for 2 h at 37°C. A microplate reader (Thermo Fisher Scientific, Waltham, MA, USA) was used to measure the absorbance value at 450 nm.

### Iron Assay

The intracellular ferrous iron (Fe2^+^) concentration was determined by colorimetric kit (cat: E-BC-K139-M, Elabscience, Wuhan, China) in accordance with the manufacturer’s protocol. Briefly, the cellular supernatant was harvested from U251 and U87 cells in different groups. Afterward, 75 μl samples in the tube were incubated with 300 μl iron chromogenic agent and 20 μl supernatant was obtained *via* a 10-min centrifugation (3,000×g, 4°C). Next, the sample values were calculated according to the optical density value of absorbance at 530 nm.

### Measurements of ROS

We determined cellular ROS production using a DCFDA/H2DCFDA-Cellular ROS Assay Kit (ab113851, Abcam, USA) in accordance with the manufacturer’s instructions. In brief, U87 and U251 cells at a density of 1 × 10^5^ cells per well from different groups were plated into six-well plates and cultured overnight. The next day, after washing three times with PBS, cells were incubated with 10 μM DCFH-DA at 37°C for 30 min. Subsequently, flow cytometry (FACSCalibur, BD, Franklin Lakes, NJ, USA) was used to monitor the fluorescence of cells and the amount of intracellular ROS was calculated by analyzing the fluorescence intensity.

### RNA Isolation and Quantitative Real-Time PCR

Total RNA was isolated using TRIzol^®^ reagent (Invitrogen). 1 μg total RNA was reverse-transcribed into cDNA using GoScript Reverse Transcriptase (A5001, Promega). QPCR analyses were performed using SYBR Premix Ex Taq II (Takara, Dalian, China) and a LightCycler^®^ 480 Real-Time PCR System (Roche, Basel, Switzerland). The expression levels were calculated using the 2^-△△^ *^C^* ^t^ method, and GAPDH was used as the internal standard. The primers of ATF4 were as follows: forward primer: 5′-TCCGCAGGCCACAAATCA-3′, reverse primer: 5′-GTCTCGGGTCGCTGCTAGT-3′. The primers of GAPDH were as follows: forward primer: 5′-GGTGAAGGTCGGAGTCAACG-3′, reverse primer: 5′-CAAAGTTGTCATGGATGACC-3′. All assays were performed in triplicate.

### Whole-Transcriptome Sequencing (RNA-Seq)

Total RNA was isolated, and ribosomal RNA was depleted. Strand-specific adapters were added to fragmented RNA (average fragment length 200 nt) before reverse transcription followed the manufacturer’s instructions. The quality of cDNA libraries was quality evaluated on an Agilent 2100 Bioanalyzer and sequenced by Shanghai Genergy Co., Ltd. (Shanghai, China). Samples were on an Illumina HiSeq 3000 platform for 2 × 150-bp paired-end sequencing. The threshold values of differentially expressed mRNA and lncRNAs were set by log_2_FoldChange > 1 and p-value < 0.05.

### Bioinformatics Analysis

The differentially expressed genes after Sev treatment were assigned to the Gene Ontology (GO) terms (http://www.geneontology.org/). The biological pathway was analyzed by searching the Kyoto Encyclopedia of Genes (KEGG) database (http://www.genome.ad.jp/kegg/). Gene set enrichment analysis (GSEA) was performed to analyze the association between the risk score of pathways and the hallmarks by GSEA Java software v4.0.3. The data were divided into two groups (control and Sev group) based on the risk score (low and high).

### Western Blotting Analysis

After harvesting glioma cells from different groups, we extracted all protein samples from cells using ice-cold RIPA lysis buffer (Beyotime Institute of Biotechnology, Shanghai, China). After BCA assay (P0012, Beyotime) for protein quantification, an equal amount of protein sample (30 μg) was separated by 8%–12% SDS-PAGE gel and then transferred onto PVDF membranes (Millipore, Burlington, MA, USA). The membranes were blocked with 5% non-fat milk dissolved in TBST for 2 h at room temperature, which were further incubated overnight with primary antibodies against ATF4 (1:500), CHAC1 (1:500), GPX4 (1:1,000), transferrin (1:500), ferritin (1:500), Beclin-1 (1:1,000), HSP70 (1:1,000), and GAPDH (1:2,000) at 4°C, followed by incubation for 2 h with an HPR-labeled secondary antibody at room temperature. Finally, the immunoblots were visualized by enhanced chemiluminescence with GAPDH or HSP70 as the loading control.

### Statistical Analysis

All quantitative data presented as mean ± standard deviation of three independent experiments were analyzed by SPSS version 19.0 (SPSS Inc., Chicago, IL, USA). Differences for two groups were evaluated using Student’s t-test and for more than two groups were assessed by one-way analysis of variance followed by Dunnett’s test or Tukey’s test. All p-values less than 0.05 were thought as statistically significant differences.

## Results

### Sev Promoted the Accumulation of Ferroptosis-Associated Iron and ROS in Glioma Cells in a Dose-Dependent Manner

**Figure 1:**
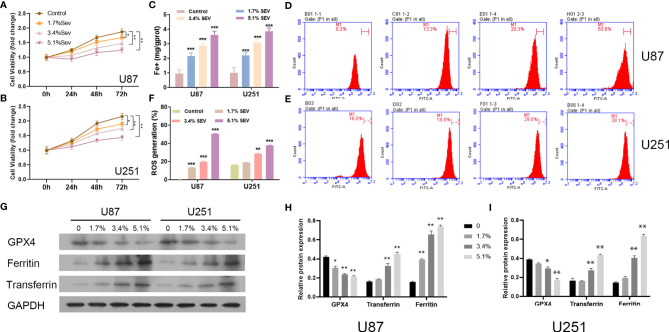
Dose-dependent effects of Sev on ferroptosis in glioma cells. U87 and U251 cells were treated with 1.7%, 3.4%, and 5.1% Sev, respectively. Cell viability was detected using CCK-8 assay in Sev-treated U87 **(A)** and U251 cells **(B)**. Fe^2+^ concentrations were determined by colorimetric assay in Sev-treated U87 and U251 cells **(C)**. ROS assay combined with flow cytometry was used to observe the content of ROS generation in SEV-treated U87 **(D)** and U251 cells **(E)**; the ratio of ROS generation was calculated **(F)**. The expression of ferroptosis-associated protein GPX4, ferritin, and transferrin in U87 and U251 cells was detected using Western blotting; GAPDH was used as the internal control **(G, H, I)**. Data were expressed as mean ± standard deviation. *p < 0.05, **p < 0.01, ***p < 0.001, compared with control.

Here, we investigated whether Sev could induce ferroptosis in glioma cells with different concentrations. According to the data from the CCK-8 assay, Sev treatment could significantly suppress U87 and U251 cell viability in a dose-dependent manner (**Figures 1A, B**). Then, we observed that treatment with Sev in a dose-dependent manner significantly increased the levels of Fe^2+^ in both U87 and U251 cells (**Figure 1C**). Moreover, with the increasing concentration of Sev, the levels of ROS generation in both U87 and U251 cells were remarkably elevated (**Figures 1D**–**F**). We additionally observed that Sev decreased the expression of ferroptosis-associated protein GPX4 and increased the expression of ferritin and transferrin in a dose-dependent manner (**Figures 1G**–**I**).

### Sev Regulated Gene Transcriptional Levels in Glioma Cells

**Figure 2:**
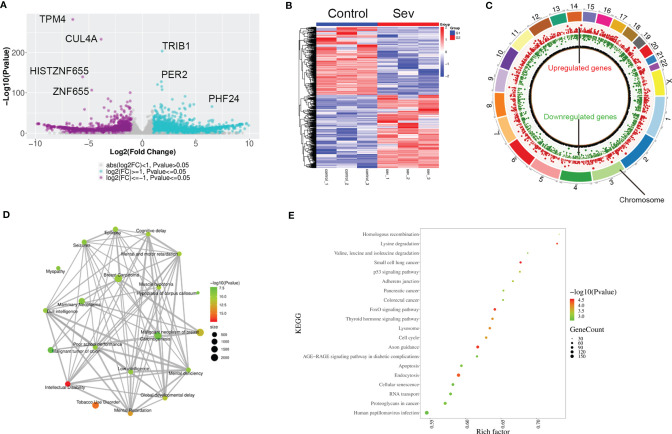
Profiling of RNAs in Sev-treated U251 cells. **(A)** The volcano figure showing the variation in 5.1% Sev-treated U251 cells and the control. The purple plot indicates RNAs with greater than 2.0-fold upregulation between the two compared groups, while the blue plot indicates the downregulated RNAs. **(B)** A heat map showing the upregulated and downregulated genes in Sev-treated U251 cells compared to the control. Each row corresponds to an RNA, each line to a sample. A Higher expression level is indicated by red, and lower level is indicated by blue. **(C)** The distribution of differentially expressed RNAs on chromosomes. The outer circle indicated each chromosome, the red inner circle indicated upregulated genes and their fold change, and the green inner circle indicated downregulated genes and their fold change. **(D)** GO analysis of significantly dysregulated genes. **(E)** KEGG pathway analysis of significantly dysregulated genes.

To explore the possible mechanisms of Sev-inducing ferroptosis in glioma cells, we performed transcriptional sequencing to screen differentially expressed genes in Sev-treated U251 cells. Sev upregulated 4,519 mRNA and long non-coding RNA (lncRNA) expression and downregulated 3,870 mRNA and lncRNA expression in U251 cells (**Figures 2A, B**). These Sev-regulated RNAs were distributed in every chromosome (**Figure 2C**). The genes regulated by Sev mainly participated in malignant neoplasm of breast, carcinogenesis, mammary neoplasms, breast carcinoma, dull intelligence, poor school performance, low intelligence, mental deficiency, intellectual disability, global developmental delay, epilepsy, seizures, mental and motor retardation, and myopathy, revealed by GO analysis (**Figure 2D**). The differential genes were involved in multiple pathways including small cell lung cancer, p53 signaling pathway, pancreatic cancer, colorectal cancer, FoxO signaling pathway, lysosome, cell cycle, apoptosis, endocytosis, cellular senescence, and proteoglycans in cancer, revealed by KEGG analysis (**Figure 2E**).

### Sev Increased Ferroptosis-Related Gene ATF4 Expression in Glioma Cells

**Figure 3:**
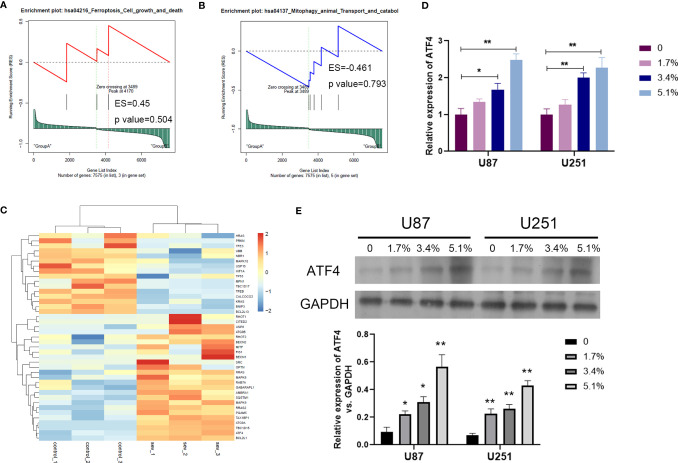
Sev upregulated ferroptosis-associated gene ATF4 in glioma cells. **(A)** Representative GSEA plot depicting ferroptosis involved in Sev-treated U251 cells. **(B)** Representative GSEA plot depicting mitophagy involved in Sev-treated U251 cells. **(C)** A heat map showing the upregulated and downregulated genes involved in ferroptosis and mitophagy in Sev-treated U251 cells compared to the control. Each row corresponds to a RNA, each line to a sample. Higher expression level is indicated by yellow, and lower level is indicated by blue. **(D)** The mRNA levels of ATF4 in Sev-treated U87 and U251 cells were detected by qRT-PCR. **(E)** The protein level of ATF4 in Sev-treated U87 and U251 cells was detected by Western blotting. Data were expressed as mean ± standard deviation. *p < 0.05, **p < 0.01, compared with control, GAPDH is used as internal control.

In order to clarify the mechanism of Sev on ferroptosis in glioma cells, we performed GSEA analysis to screen the differential pathway and related gene expression. Sev could regulate a few ferroptosis and mitophagy-associated genes identified by GSEA analysis (**Figures 3A, B**). Sixteen genes were downregulated and 25 genes were upregulated by Sev in U251 cells. In these differentially expressed genes, the expression of activating transcription factor 4 (ATF4) was the second upregulated gene (**Figure 3C**). To analyze whether Sev enhanced the expression of ATF4, we performed qRT-PCR and Western blotting assay. The data indicated that the mRNA and protein levels of ATF4 were enhanced by Sev in a dose-dependent manner (**Figures 3D, E**). These results suggested that Sev induces ferroptosis *via* activating ATF4 in glioma cells.

### Sev Induced Ferroptosis by ATF4 in Glioma Cells

**Figure 4:**
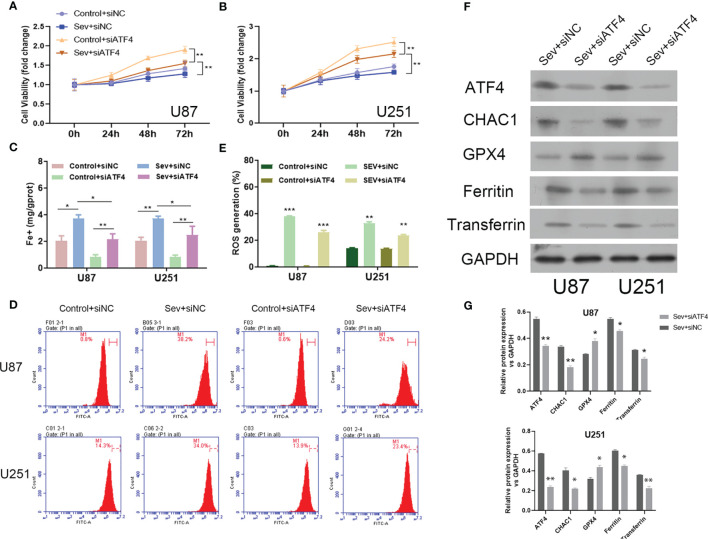
The effects of ATF4 on Sev-induced ferroptosis in glioma cells. U87 and U251 cells were transfected with ATF4 siRNA or negative control sequences, followed by treatment with 5.1% Sev for 2 h. Cell viability was detected using CCK-8 assay in U87 **(A)** and U251 cells **(B)**. Fe2+ concentrations were determined by colorimetric assay in U87 and U251 cells **(C)**. ROS assay combined with flow cytometry was used to observe the content of ROS generation in U87 and U251 cells **(D)**; the ratio of ROS generation was calculated **(E)**. The expression of ferroptosis-associated protein CHAC1, GPX4, ferritin, and transferrin in U87 and U251 cells was detected using Western blotting; GAPDH was used as the internal control **(F, G)**. Data were expressed as mean ± standard deviation. *p < 0.05, **p < 0.01, ***P<0.0001, compared with control.

We firstly observed that inhibition of ATF4 significantly attenuated Sev-mediated suppressive effects on U87 and U251 cell viability (**Figures 4A, B**). Secondly, the elevated Fe^2+^ concentration by Sev treatment in both U87 and U251 cells was strongly suppressed by ATF4 suppression (**Figure 4C**). Thirdly, the ROS accumulation induced by Sev treatment in U87 and U251 cells was impaired after ATF4 suppression (**Figures 4D, E**). At the molecular level, we further found that inhibition of ATF4 obviously reversed the effects of Sev on protein levels of GPX4, transferrin, and ferritin. Moreover, ChaC glutathione-specific gamma-glutamylcyclotransferase 1 (CHAC1), which is involved in ferroptosis, is a downstream gene of ATF4. We also verified that inhibition of ATF4 could interrupt the expression of CHAC1 induced by Sev in U87 and U251 cells (**Figures 4F, G**). These results indicated that Sev regulated ferroptosis by modulating ATF4-CHAC1 pathway in glioma cells.

### Induction of Ferroptosis Reversed ATF4 Suppression-Mediated Effects on Sev-Promoted Ferroptosis

**Figure 5:**
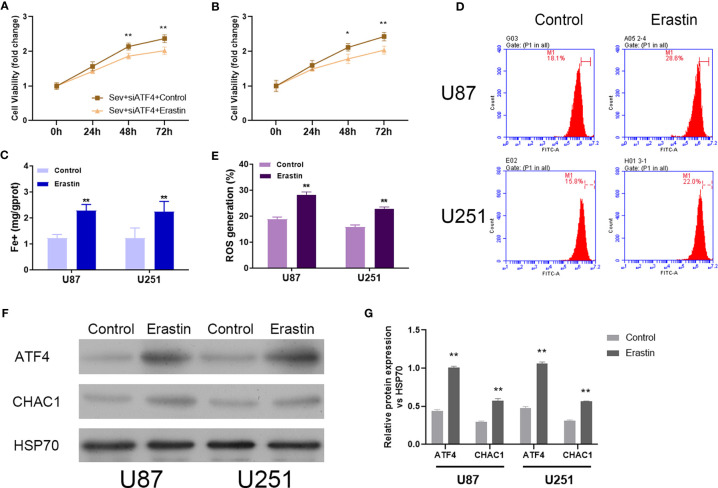
Induction of ferroptosis reversed the effects of ATF4 suppression on Sev-induced ferroptosis in glioma cells. Ferroptosis inducer Erastin was used to incubate in Sev-treated and ATF4-suppressed U87 and U251 cells. Cell viability was detected using CCK-8 assay in U87 **(A)** and U251 cells **(B)**. Fe2+ concentrations were determined by colorimetric assay in U87 and U251 cells **(C)**. ROS assay combined with flow cytometry was used to observe the content of ROS generation in U87 and U251 cells **(D)**; the ratio of ROS generation was calculated **(E)**. The expression of ATF4 and CHAC1 in U87 and U251 cells was detected using Western blotting; GAPDH was used as the internal control **(F, G)**. Data were expressed as mean ± standard deviation. *p < 0.05, **p < 0.01, compared with control.

To further confirm the regulatory role of Sev on ferroptosis by modulating ATF4 in glioma cells, ferroptosis inducer Erastin was utilized to incubate Sev and ATF4 siRNA-co-treated U87 and U251 cells. Our data showed that Erastin treatment obviously inhibited the cell viability (**Figures 5A, B**), elevated the Fe^2+^ concentration (**Figure 5C**), and promoted ROS generation (**Figures 5D, E**) in U87 and U251 cells. Moreover, in the Erastin treatment group, the protein level of ATF4 and CHAC1 was increased in U87 and U251 cells (**Figures 5F, G**). These data indicated that ferroptosis inducer Erastin could restore the Sev-induced ATF4-CHAC1 pathway activity which was restrained by ATF4 siRNA in glioma cells.

## Discussion

Here, we firstly observed that Sev treatment promoted ferroptosis in glioma cells in a dose-dependent manner. It is well accepted that ferroptosis is a form of cell death dependent on iron correlated with oxidative damage with main features including lipid ROS accumulation, loss of mitochondrial cristae, and an increase in mitochondrial membrane density (11, 29). Ferroptosis is reported to be initiated with the loss of GPX4. Then the loss of GPX4 induces the accumulation of peroxides in the membrane which results in aggregation of destructive lipid ROS (30). In fact, Sev as an inhalational anesthetic has been widely reported its suppressive role in glioma cell proliferation and invasion through regulating different pathways, such as the miR-124-3p/ROCK1 axis (24), miR-146-5p/MMP16 (31), miR-628-5p/MAGT1 axis (27), and PI3K/AKT signaling pathway (32). Here, we identified another function of Sev on suppressing glioma cell proliferation *via* activation of ferroptosis. Even though Sev was reported to inhibit ferroptosis and to exhibit a protective role against lipopolysaccharide-induced acute lung injury (33), the effect of Sev on ferroptosis of glioma cells was the first time to be illustrated in this study.

We further demonstrated that Sev treatment suppressed the expression of GPX4, while upregulating the expression of transferrin and ferritin and Beclin-1 in glioma cells in a dose-dependent manner. GPX4, an essential regulator of ferroptosis, and its expression level are inversely correlated with ferroptosis regulation by decreasing ROS productions or cellular iron (34). In addition, transferrin and ferritin are well-known positive regulators of cellular iron (35), which make it easy to explain their upregulation after Sev-induced ferroptosis. Our results demonstrated that Sev-induced ferroptosis in glioma cells was in a GPX4-dependent pathway.

In order to illustrate the mechanisms of Sev-inducing ferroptosis in glioma cells, we performed transcriptional sequencing to screen differentially expressed genes and used GO, KEGG, and GSEA analyses to select enriched diseases and pathways. The data indicated that Sev could regulate a number of genes associated with several cancers and several nervous system diseases. Because Sev is an anesthetic, the target genes of Sev are possibly related to nervous system diseases. We should perform more experiments in the future to validate whether these screened genes take part in the progression of gliomas. Research has confirmed that Sev could also demonstrate anticancer effects in lung cancer and colorectal cancer (19, 36). Moreover, we identified that Sev functioned on ferroptosis, apoptosis, and mitophagy *via* analyzing transcriptional sequencing data. Sev was reported to promote apoptosis in colon cancer (37), lung cancer (38), and ovarian cancer (39). Even the function of Sev on ferroptosis and mitophagy in cancers has not been reported, although some research demonstrated that Sev induced ferroptosis and mitophagy in other disease models. Exposure of Sev in young age mice was found to trigger mitophagy (40). Zhao et al. performed an *in vivo* experiment and certified that knockdown of neuronal regulator MIB2 could alleviate ferroptosis of neuron induced by Sev exposure (41). Our results and other studies indicated that Sev could induce ferroptosis in multiple diseases including glioma. Besides mRNAs, the results of RNA-seq also discovered that Sev could regulate the expression of few lncRNAs in glioma cells. LncRNAs are non-coding RNAs longer than 200 nt, which can interact with RNA, proteins, and DNA. Deregulation of lncRNAs take part in the initial and progression of gliomas (42). He et al. established a 14-lncRNA panel related to ferroptosis, tumor progression, and microenvironment to predict the prognosis of gliomas (43). Several lncRNAs were found to be associated with ferroptosis in gliomas. LncRNA TMEM161B-AS1 was identified to promote the malignant behavior and temozolomide resistance through enhancing the expression of multiple ferroptosis-related genes (44). However, we did not validate the expression of lncRNAs screened by RNA-seq using experimental methods. Since the functions and mechanisms of lncRNAs in gliomas are hot topics in the recent years, it is very meaningful to explore the function and mechanism of Sev on regulating lncRNAs in glioma cells in the future.

In order to reveal the exact pathway of ferroptosis induced by Sev in glioma cells, we selected differentially expressed genes and found that the fold change of ATF4 was the second increased. ATF4 is an essential regulator of endoplasmic reticulum stress and recently found to be a mediator of ferroptosis. ATF4 has a dual role for cell death. On the one hand, ATF4 is required for long-term survival by controlling the expression of genes involved in metabolism and protection from oxidative stress. On the other hand, ATF4 can also trigger apoptosis, cell-cycle arrest, ferroptosis, and senescence (45). In tumor cells, ATF4 demonstrates contrary functions in different conditions. In some studies, suppression of ATF4 could inhibit cell proliferation and metastasis of glioma cells. Upregulation of miR-1283 suppresses the progression of glioma cells by targeting ATF4 (46). Silencing P2X4R interrupts the proliferation of glioma cells by downregulating the BDNF/TrkB/ATF4 pathway (47). Dihydroartemisinin could attenuate ferroptosis *via* the PERK/ATF4/HSPA5 pathway in glioma cells (48). However, in other research, activating ATF4-dependent pathways produces anticancer effects. Ergul et al. identified that thiamine protected glioblastoma cells against glutamate toxicity by inhibiting ATF4-associated endoplasmic reticulum stress (49). Recently, a TRAIL-inducing compound ONC201/TIC10 was found to promote apoptosis and integrated stress response in multiple tumors by activating ATF4 (50). Overexpression of ATF4 is suggested to enhance the susceptibility of cancer cells to ferroptosis (51). Ferroptotic agents were found to activate ER stress response (52). Evidence showed that the ferroptotic agent ART enhances the expression of ATF4-downstream genes (53). Withaferin A was found to induce G2/M arrest and apoptosis in glioblastoma cells through activating the ATF4-ATF3-CHOP pathway (54). Under Sev treatment, we also observed that ATF4 protein levels in U87 and U251 cells were significantly increased. Through rescue experiments, we demonstrated that inhibition of ATF4 attenuated Sev-mediated cell proliferation, iron accumulation, and ROS generation. Moreover, ATF4 suppression obviously reversed the regulatory role of Sev on protein levels of GPX4, transferrin, and ferritin in glioma cells. To further confirm the activation of ferroptosis under Sev-treated glioma cells, ferroptosis inducer Erastin was used to incubate U87 and U251 cells which were treated by Sev and ATF4 siRNA. Our data showed that induction of ferroptosis reversed ATF4 suppression-mediated effects on Sev-induced proliferation, iron accumulation, and ROS accumulation in glioma cells. Our data indicated that Sev exerted activation of ferroptosis in glioma cells which was associated with promoting ATF expression. Until now, Sev exerted the ameliorative effects against ischemia-reperfusion-induced myocardial apoptosis, which might be mediated by suppressing the ATF4-correlated signaling pathway (55). Additionally, neonatal Sev exposure-induced neuroapoptosis is mediated *via* the PERK-eIF2α-ATF4-CHOP axis of the endoplasmic reticulum stress signaling pathway (56). Based on this evidence, we might speculate that Sev suppressed the proliferation of glioma cells by ATF4-mediated ferroptosis. The reason of ATF4 playing a dual role in glioma is unclear. The possible mechanism may be epigenetic modification (45).

Considering that ATF4 transcriptionally regulates multiple genes associated with cancer, we planned to explore Sev-associated ATF4-regulated genes in glioma cell ferroptosis. Chen et al. reported that degradation of glutathione by CHAC1 induced necroptosis and ferroptosis in human triple-negative breast cancer cells *via* a ATF4-dependent manner (57). CHAC1 is a pro-apoptotic gene with enzymatic activity of glutathione-specific γ-glutamyl cyclotransferase. CHAC1 can be activated by the ATF4-CHOP axis at the transcriptional level (58, 59). Activation of the ATF4-CHOP-CHAC1 pathway promotes ferroptosis in Burkitt’s lymphoma DAUDI and CA-46 cells (60). In glioma cells, CHAC1 can bind to Notch3 protein and inhibit its activation under temozolomide induction, leading to inactivation of Notch3-mediated downstream signaling pathways (61). In our study, Sev induced the expression of CHAC1 in glioma cells, and this effect could be attenuated by inhibiting ATF4 expression. According to these results, there may be several interactive mechanisms between ATF4 and CHAC1. Based on the published references, ATF4 upregulated CHAC1 at the transcriptional level. However, the mechanism of CHAC1 regulating ATF4 was unknown. In the future research, we should investigate whether ATF4 could bind to CHAC1 directly; if they bind to each other, the area in genes or proteins is the binding sites.

In summary, our data manifested that Sev suppressed the proliferation of glioma cells at least in part by activating ferroptosis *via* upregulating the ATF4–CHAC1 pathway. The results provided a novel mechanism that Sev induced ferroptosis in glioma cells, which is possibly a potential therapeutic target for glioma treatment.

## Conclusion

Sev suppressed the proliferation of glioma cells at least in part by activating ferroptosis *via* upregulating the ATF4–CHAC1 pathway.

## Data Availability Statement

The datasets presented in this study can be found in online repositories. The names of the repository/repositories and accession number(s) can be found in the following: https://www.ncbi.nlm.nih.gov/geo/query/acc.cgi?acc=GSE193295.

## Author Contributions

YX and XL contributed to the conception and design of the study. YX and NZ wrote the first draft of the manuscript. CC analyzed the results of RNA-seq. XX performed the cell culture; AL performed the CCK8 and Western blotting. YY conducted the qRT-PCR and flow cytometry. YhL, JL, and XO treated cells with sevoflurane. YfL measured the concentration of iron and ROS. YT and LC performed the statistical analysis. XS and XL revised the manuscript. All authors contributed to the manuscript revision and read and approved the submitted version.

## Funding

The Science and Education Projects of Guangzhou Health Commission (20201A010019) supported YfL. The Basic and Applied Basic Research Foundation of Guangdong Province (2020A1515110475) supported YX. The Science and Technology Projects in Guangzhou (202102080226) supported YfL.

## Conflict of Interest

The authors declare that the research was conducted in the absence of any commercial or financial relationships that could be construed as a potential conflict of interest.

## Publisher’s Note

All claims expressed in this article are solely those of the authors and do not necessarily represent those of their affiliated organizations, or those of the publisher, the editors and the reviewers. Any product that may be evaluated in this article, or claim that may be made by its manufacturer, is not guaranteed or endorsed by the publisher.
